# Supplementary material for: Does Eating-Away-from-Home Increase the Risk of a Metabolic Syndrome Diagnosis?
Source: Int J Environ Res Public Health. 2019 Feb 16;16(4):575. doi: 10.3390/ijerph16040575 (PMC6406498; doi:10.3390/ijerph16040575)
Supplement: Supplementary file 1 [file ijerph-16-00575-s001.pdf]

Supplementary file

Table S1 Multivariable adjusted[1] association between Mets and EAFH

	Total population (n=4518)	Female (n=2441)	Male (n=2077)
EAFH frequency	1.104(1.049, 1.162)	0.972(0.911, 1.037)	1.083(1.027, 1.141)
Female	1.143(0.914, 1.430)		
Female*fafh	0.859(0.796, 0.928)		
Age	1.143(1.105, 1.183)	1.174(1.114, 1.238)	1.144(1.090, 1.201)
Square of age	0.999(0.999, 0.999)	0.999(0.998, 0.999)	0.999(0.998, 0.999)

[1] Adjusted for age and age squared, educational level (primary, middle and high), ln(income), smoking (current smoking or no), drinking (current drink or no), physical activity (light, moderate and heavy), localization (urban or rural; north or south), total energy intake and fat share. For total population regression, sex was added in addition. EAFH frequency was measured by the number of meals ate away from home during the three-day period.

[2] Values are ORs (95%CI) unless otherwise indicated.

Table S2 Multivariable adjusted[1] association between Mets and EAFH by regions

	Urban (n=1398)	Rural (n=3120)	South (n=2619)	North (n=1899)
Never(referent)	1	1	1	1
Sometimes	1.466(0.920, 2.336)	1.457(1.026, 2.069)	1.648(1.145, 2.372)	1.456(0.929, 2.280)
Often	2.074(1.149, 3.743)	1.366(0.809, 2.307)	2.103(1.327, 3.331)	1.219(0.578, 2.574)
Female	1.193(0.767, 1.855)	1.145(0.875, 1.499)	1.523(1.080, 2.150)	0.946(0.692, 1.294)
Female*sometimes	0.736(0.401, 1.351)	0.579(0.350, 0.959)	0.547(0.341, 0.878)	0.565(0.289, 1.105)
Female*often	0.417(0.172, 1.008)	0.521(0.232, 1.173)	0.310(0.150, 0.642)	0.708(0.249, 2.012)
Middle aged	2.523(1.767, 3.604)	1.757(1.415, 2.181)	2.062(1.581, 2.688)	1.838(1.423, 2.374)
Elderly adults	2.634(1.679, 4.133)	1.651(1.244, 2.190)	2.187(1.587, 3.014)	1.553(1.087, 2.218)

[1] Adjusted for age and age squared, educational level (primary, middle and high), ln(income), smoking (current smoking or no), drinking (current drink or no), physical activity (light, moderate and heavy), localization (urban or rural; north or south), total energy intake and fat share. For total population regression, regional dummy was added in addition. Never, sometimes, and often refer to the frequency of EAFH during the three-day period: never (0), sometimes (>0 and <=3), and often (>3 and <=12).

[2] Values are ORs (95%CI) unless otherwise indicated.

Table S3 Frequency of various meals EAFH by age and gender

	Young				Middle aged				Old			
	Female(n=987)		Male (n=819)		Female (n=937)		Male (n=757)		Female (n=517)		Male (n=501)	
Meals	Mean	S. D.	Mean	S. D.	Mean	S. D.	Mean	S. D.	Mean	S. D.	Mean	S. D.
break_away	0.42	0.95	0.49	1.01	0.29	0.84	0.41	0.96	0.24	0.75	0.21	0.69
lunch_away	0.48	1.00	0.50	1.01	0.21	0.67	0.44	0.96	0.11	0.49	0.14	0.57
dinner_away	0.21	0.66	0.22	0.67	0.10	0.44	0.19	0.63	0.08	0.41	0.07	0.40
snack_away	0.11	0.50	0.09	0.46	0.08	0.45	0.11	0.52	0.06	0.34	0.07	0.42
Frequency	1.23	2.23	1.30	2.24	0.68	1.61	1.15	2.13	0.49	1.34	0.49	1.41
Share_EAFH	12.21%	0.22	13.47%	0.23	6.82%	0.16	11.84%	0.22	5.03%	0.14	5.01%	0.14

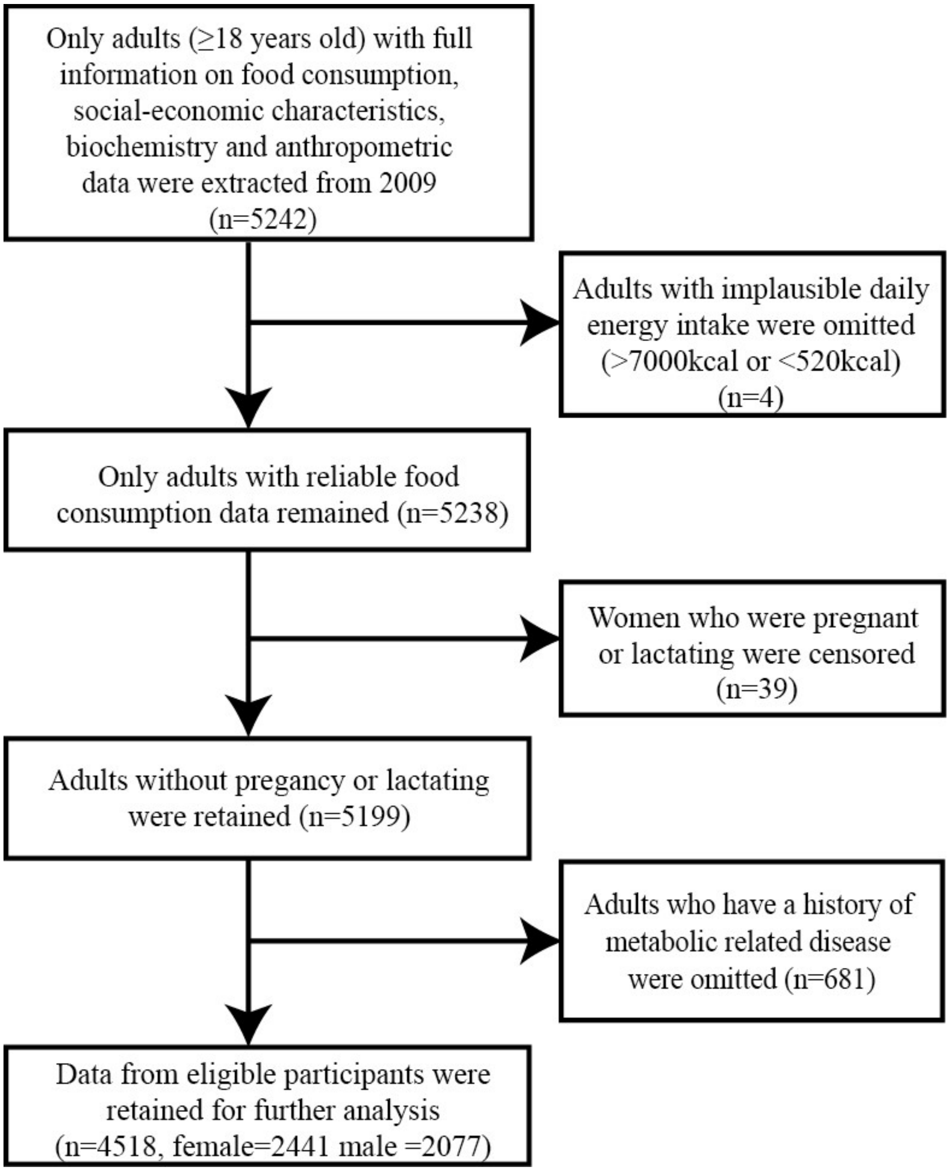

Figure S1 Selection of sample in the study

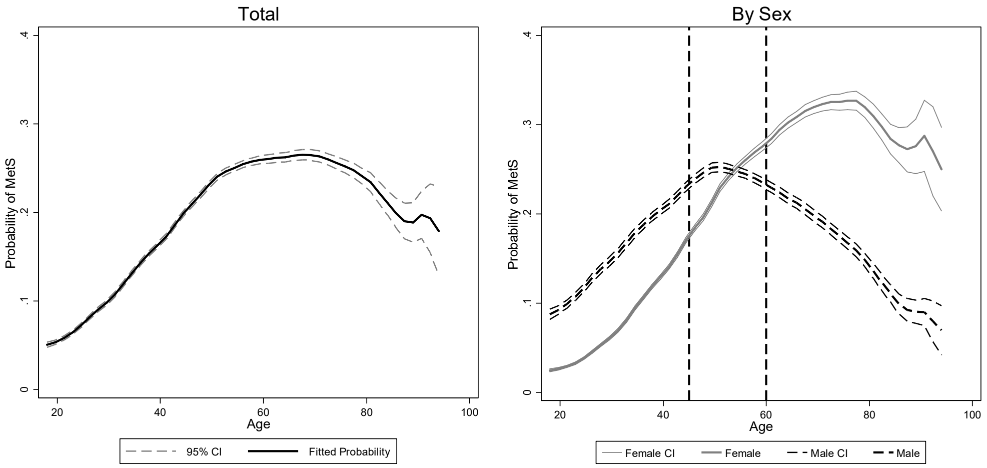

Figure S2 Association between predicted risk of getting metabolic syndrome and age
